# Fecal Keratin 8 Is a Noninvasive and Specific Marker for Intestinal Injury in Necrotizing Enterocolitis

**DOI:** 10.1155/2023/5356646

**Published:** 2023-03-14

**Authors:** Kewei Wang, Guozhong Tao, Zhen Sun, Jingjing Wei, Junlin Liu, Jordan Taylor, Michelle Gibson, Mirko Mostaghimi, Misty Good, Karl G. Sylvester

**Affiliations:** ^1^Department of Gastrointestinal Surgery, The First Hospital of China Medical University, Shenyang 110001, China; ^2^Department of Surgery, Stanford University School of Medicine, Stanford, CA 94305, USA; ^3^Stanford Metabolic Health Center, Stanford University School of Medicine and Stanford Healthcare, Stanford, CA 94305, USA; ^4^Department of Pediatrics, Pathology and Immunology Division of Newborn Medicine, Washington University School of Medicine, St. Louis Children's Hospital, St. Louis, MO 63110, USA

## Abstract

Specific biomarkers of intestinal injury associated with necrotizing enterocolitis (NEC) are needed to diagnose and monitor intestinal mucosal injury and recovery. This study aims to develop and test a modified enzyme-linked immunosorbent assay (ELISA) protocol to detect the total keratin 8 (K8) in the stool of newborns with NEC and investigate the clinical value of fecal K8 as a marker of intestinal injury specifically associated with NEC. We collected fecal samples from five newborns with NEC and five gestational age-matched premature neonates without NEC at the Lucile Packard Children's Hospital Stanford and Washington University School of Medicine, respectively. Fecal K8 levels were measured using a modified ELISA protocol and Western blot, and fecal calprotectin was measured using a commercial ELISA kit. Clinical data, including gestational age, birth weight, Bell stage for NEC, feeding strategies, total white blood cell (WBC) count, and other pertinent clinical variables, were collected and analyzed. Fecal K8 levels were significantly higher in the pre-NEC group (1–2 days before diagnosis of NEC) and NEC group than those in the non-NEC group (*p* = 0.013, *p* = 0.041). Moreover, fecal K8 was relatively higher at the onset of NEC and declined after the resolution of the disease (*p* = 0.019). Results with similar trends to fecal K8 were also seen in fecal calprotectin (*p* = 0.046), but not seen in total WBC count (*p* = 0.182). In conclusion, a modified ELISA protocol for the total K8 protein was successfully developed for the detection of fecal K8 in the clinical setting of premature newborns with NEC. Fecal K8 is noted to be significantly increased in premature newborns with NEC and may, therefore, serve as a noninvasive and specific marker for intestinal epithelial injury associated with NEC.

## 1. Introduction

Necrotizing enterocolitis (NEC) is one of the leading causes of severe morbidity and mortality in affected preterm neonates [1, 2]. The clinical manifestations of NEC can progress over a short period from nonspecific gastrointestinal (GI) signs including abdominal distension and feeding intolerance to rapid systemic deterioration including multiple organ dysfunction and shock within several hours [3]. Diagnosis of NEC is based on the combination of clinical, laboratory, and radiologic findings. Abdominal radiography is one of the most critical modalities for securing a diagnosis given the pathognomonic findings of intestinal wall pneumatosis and portal venous gas that indicate significant enteric epithelial compromise [4]. However, the challenge is significant when there is discordance between clinical symptoms and abdominal radiographic findings. Moreover, a newborn's clinical symptoms and imaging findings may appear late in the course of the disease, leading to a delay in treatment and a potentially narrow therapeutic window to prevent disease progression. Although several potential fecal markers that may be used to predict or diagnose NEC have been identified in recent years, in general, the specificity of most candidate biomarkers to date remains to be improved [5–7]. The primary pathological manifestation of NEC is intestinal epithelial injury. Following intestinal epithelial injury, enteric epithelial cells undergo death and detachment from the intestinal wall, mixing and excreting with the fecal stream. Therefore, we hypothesized that the detection of epithelial cell-specific cellular constituents in feces would provide a direct and highly specific method to detect the enteric mucosal injury associated with NEC.

Keratin 8 (K8) is an abundant cytoskeletal protein, which forms intermediate filament (IF) networks together with K18/K19/K20 within a simple or single-layered epithelia, including enterocytes [8], hepatocytes [9], etc. Like all other keratins, K8 has high stability and low solubility (∼5% of K8 is soluble) [10]. Routine sandwich enzyme-linked immunosorbent assay (ELISA) is not suitable for insoluble protein measurement. This study aims to develop and test a modified ELISA protocol to detect the total IF protein K8 in NEC neonatal feces and investigate the potential clinical specificity of fecal K8 as a marker of intestinal damage in NEC.

## 2. Materials and Methods

### 2.1. Fecal Samples

Fecal samples were obtained from five neonates with NEC at Lucile Packard Children's Hospital Stanford at different time points. To eliminate the possible interference of different gestational ages (GA) on K8 detection results, we also collected fecal samples from five GA-matched non-NEC neonates as the control (named Ctrl1). All neonatal fecal samples were stored at −80°C for later use. Before detection, the samples were thawed at 4°C. Fecal samples of GA-matched control group were chosen specially at a similar day of life (DOL) to match fecal samples of NEC infants at the time of disease onset. Clinical data included GA, sex, birth weight, DOL at NEC/control sampling, modified Bell stage, feeding strategies, length of neonatal intensive care unit (NICU) stay, and white blood cell (WBC) count.

In addition, serial fecal samples of premature neonates with a high risk of NEC were collected from birth admitted to the NICU at the Washington University School of Medicine in St. Louis. Five neonates were diagnosed with NEC, and their fecal samples about 2 days before a definitive diagnosis of NEC were collected for analysis. Feces samples of GA-matched control group (named Ctrl2) were chosen specially at the similar DOL to match those of infants with NEC at the time of disease onset.

This study was approved by the Institutional Review Board of Stanford University. Written informed consent was obtained from the parents of all neonates for stool sampling.

### 2.2. Modified ELISA Protocol to Measure the Total K8 Protein in Stool (Figures 1(a) and 1(b))

Ninety-six-well MaxiSorp™ Nunc-immuno module plates (Thermo Fisher Scientific, Waltham, MA, USA) were coated with 10 *μ*g/ml HT29 cell lysates (ATCC, Manassas, VA, USA) containing antigen K8 in coating buffer (50 *μ*l/well) overnight at 4°C. Twenty-five microliter of diluted samples or K8 standards (Abcam, USA) were mixed with 25 *μ*l of blocking buffer (5% skim milk/PBST/1% NP40) for 30 min with shaking. Fifty microliter of anti-keratin 8 antibodies (Troma-1, MilliporeSigma, Temecula, CA, USA, 1 : 8,000) were added to each tube and incubated at room temperature (RT) for 1 hr with shaking. The precoated plates were washed three times with PBS buffer. Premixed samples with antibodies were transferred to each well and incubated at RT with shaking for 1 hr (100 *μ*l/well), followed by washing three times with PBST buffer. One-hundred microliter of horseradish peroxidase (HRP)-conjugated goat anti-rat antibodies (ImmunoReagents Inc., Raleigh, NC, USA) diluted 1 : 5,000 with PBST/5% skim milk buffer were added to each well and incubated at RT with shaking for 30 min. Plates were washed five times and visualized by incubating in a TMB substrate kit (Thermo Fisher Scientific, 100 *μ*l/well) for 15 min. One-hundred microliter of 2 M H_2_SO_4_ was added to each well to stop color development. The plates were read at 450 nm with a reference filter at 620 nm using the SpectraMax® i3x Multi-Mode Microplate Reader (Molecular Devices, CA, USA).

### 2.3. Calculation of K8 Concentration

**Figure 1:**
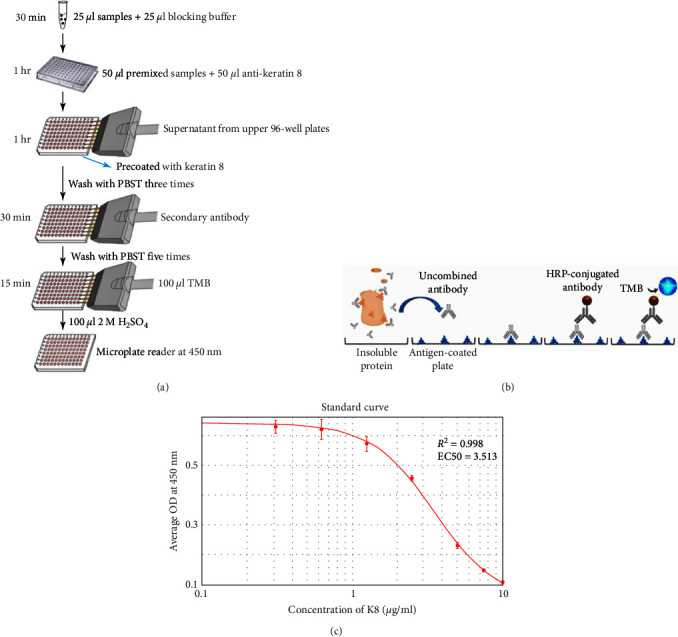
Modified ELISA protocol to measure total K8 proteins in the stool: (a) particular operating procedures. The blocking buffer is composed of 5% skim milk, PBST, and 1% NP40. PBST, phosphate-buffered saline with Tween 20; TMB, tetramethylbenzidine; and H_2_SO_4_, sulfuric acid; (b) schematic diagram of the principle. Individual fecal samples were diluted to a suitable level in PBS/EDTA buffer within the measurement range of the assay. We configured a new protocol by measuring the remaining amount of free unbound primary antibody after antigen–antibody reaction. K8 levels were calculated using 4-parameter nonlinear logistic curve fitting (4-PL) to assess the total level of both insoluble and soluble K8; (c) standard curve of K8 (mean ± SD). The plates, as shown in Figure 1(a), were read at 450 nm with a reference filter at 620 nm using the SpectraMax® i3x Multi-Mode Microplate Reader. OD, optical density; K8, keratin 8. Note that a routine sandwich ELISA cannot measure the insoluble K8, which may account for 95% of the total amount. The new ELISA protocol can assess total fecal K8 with the majority of insoluble forms.

An eight-point standard curve of K8 ranging from 0 to 10 *μ*g/ml was constructed to analyze the level of K8 in fecal samples (Figure 1(c)). Individual fecal samples were diluted to a suitable level in PBS/EDTA buffer within the measurement range of the assay and analyzed as described above. K8 levels were calculated using 4-parameter logistic (4-PL) nonlinear curve fitting (SoftMax Pro v. 6.4.2, Molecular Devices, CA, USA). The levels measured in the diluted samples were multiplied by the dilution ratio to obtain the initial fecal K8 levels.

### 2.4. Sandwich ELISA for Fecal Calprotectin

Fecal calprotectin was measured by the commercial ELISA kit (Eagle Biosciences, Nashua, NH, USA), according to the manufacturer's instructions. The plates were read at 450 nm with a reference filter at 620 nm using the SpectraMax® i3x Multi-Mode Microplate Reader. Final concentrations were obtained using the standard curve method.

### 2.5. Western Blot (WB) Analysis for Fecal K8

An equal amount (in weight) of stool samples was mixed with RIPA lysis buffer (Thermo Fisher Scientific, Rockford, IL, USA) (0.1 g stool per ml), followed by homogenization, heat denaturation, and centrifugation, and then 10 *μ*l of the subsequent supernatants were loaded to each lane of the gel for comparison. Total proteins were separated by 10% sodium dodecyl sulfate–polyacrylamide gel electrophoresis (SDS–PAGE) gels (Bio-Rad, Hercules, CA, USA), transferred to polyvinylidene difluoride membranes (Amersham Biosciences, Buckinghamshire, UK), and incubated with primary anti-K8 (Troma-1, 1 : 2,000) overnight at 4°C. After being washed in PBST, the blots were incubated with HRP-conjugated goat anti-rat antibody (1 : 2,000) at RT for 1 hr. After a final wash as described above, protein bands were visualized using an ECL detection kit (Thermo Fisher Scientific, Rockford, IL, USA). Signals were quantified with ImageJ software (NIH.gov, USA).

### 2.6. Statistical Analysis

Categorical variables were presented as *n* (%) and statistical analysis was performed using the *χ*^2^ test. Continuous variables were presented as mean ± SD and statistical analysis was performed using Student's *t*-test (SPSS Statistics, version 19.0, Chicago, USA). *p* < 0.05 was considered as statistically significant. The data graphs were made with GraphPad Prism 5.0 software (GraphPad Software, CA, USA).

## 3. Results

### 3.1. Clinical Characteristics of Neonates

During this study period, five preterm infants from Lucile Packard Children's Hospital Stanford had a confirmed diagnosis of NEC (four with Bell stage II and one with Bell stage III) and five preterm infants were enrolled as GA- and DOL-matched controls. Five fecal samples in the NEC group were collected at NEC onset. The NEC infants had a mean GA of 29 weeks and a mean birth weight of 1,395.4 g. There were no differences in GA and birth weight between NEC and the control group (Ctrl1) (*p* = 0.76, *p* = 0.70). The mean DOL at NEC onset was day 22, which closely matched the date of sampling in the Ctrl1 group (22.2 ± 14.3 vs. 21 ± 2.3 days, *p* = 0.86). The length of NICU stay in the NEC group was significantly longer than in the Ctrl1 group (83 ± 42.9 vs. 22.4 ± 19.5 days, *p* = 0.03). Five neonates from Washington University School of Medicine had a confirmed diagnosis of NEC (two with Bell stage Ⅰ and three with Bell stage II) and five preterm infants were matched as controls (Ctrl2). Five fecal samples in a pre-NEC group were collected within 1–2 days before a diagnosis of NEC. The NEC infants had a mean GA of 27 weeks and a mean birth weight of 856.0 g. There were no significant differences in GA or birth weight between pre-NEC and Ctrl2 group (*p* = 0.53, *p* = 0.23) neonates. The mean DOL of pre-NEC samples was not significantly different compared with the Ctrl2 group (17.4 ± 5.7 vs. 10.2 ± 4.6 days, *p* = 0.06). The length of NICU stay in the pre-NEC group was longer than in the Ctrl2 group, but the difference was not statistically significant (129.6 ± 71.9 vs. 69.2 ± 28.8 days, *p* = 0.17). Neonates from both groups received parenteral nutrition. Duration of parenteral nutrition in the NEC group and the pre-NEC group was much longer than in the non-NEC group, but the difference was not statistically significant (*p* = 0.1, *p* = 0.09). The detailed clinical characteristics of neonates are summarized in Table 1.

### 3.2. Fecal K8 Levels Were Significantly Higher in the NEC Group and Pre-NEC Group than Those in the Non-NEC Group

**Figure 2:**
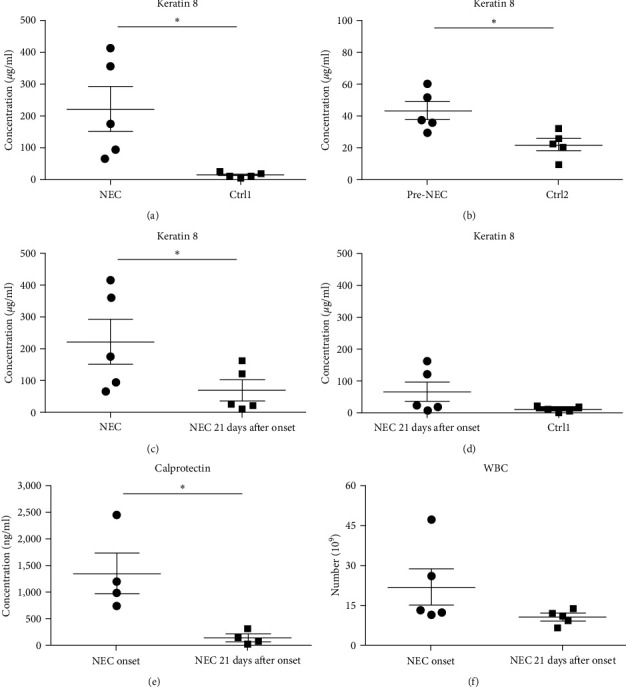
Fecal K8 levels in NEC- and GA-matched non-NEC premature neonates: (a) fecal K8 levels of neonates at initial NEC onset (i.e., when NEC was diagnosed) vs. their levels in GA-matched control group (Ctrl1) (mean ± SEM) (*n* = 5); (b) fecal K8 levels of neonates at 1–2 days before NEC were diagnosed (pre-NEC) vs. levels in GA-matched control group (Ctrl2) (*n* = 5). Note that the fecal K8 was increased ∼2 days before a clinical diagnosis, suggesting that it may become a useful biomarker for early diagnosis of NEC; (c) fecal K8 levels of neonates at initial NEC onset vs. levels at 21 days after NEC onset (*n* = 5); (d) fecal K8 levels of neonates at 21 days after NEC onset vs. levels in GA-matched control group (Ctrl1) (*n* = 5); fecal calprotectin (e) (*n* = 4) and WBC (f) (*n* = 5) at initial NEC onset vs. levels at 21 days after NEC onset. WBC, white blood cell.  ^*∗*^*p* < 0.05.

The mean fecal K8 level in the NEC group (day at onset) was 221.64 *μ*g/ml (range 65.64–413.7 *μ*g/ml), which was significantly higher compared to the GA-matched Ctrl1 group (221.64 ± 156.97 vs. 13.36 ± 7.96 *μ*g/ml, *p* = 0.041) (Figure 2(a)). The mean fecal K8 level in the pre-NEC group (1–2 days before NEC onset) was significantly higher (43.34 *μ*g/ml (range 30.04–60.62 *μ*g/ml)) compared to the GA-matched Ctrl2 group (43.34 ± 12.54 vs. 22.14 ± 8.25 *μ*g/ml, *p* = 0.013) (Figure 2(b)).

### 3.3. Fecal K8 and Calprotectin Significantly Increased in Premature Infants with NEC and Declined after Resolution of the Disease

All cases of the NEC group were noted to be resolved by the 21st day after the onset of the disease according to the symptoms, signs, and laboratory examinations. The mean fecal K8 level was 68.44 *μ*g/ml (range 10.13–162.45 *μ*g/ml) around day 21 (two samples collected on the 22nd day after NEC onset, two samples collected on the 21st day after NEC onset, and one sample collected on the 18th day after NEC onset). In every infant with NEC, fecal K8 was significantly higher at the onset of NEC and declined after the resolution of the disease (221.64 ± 156.97 vs. 68.44 ± 69.54 *μ*g/ml, *p* = 0.019) (Figure 2(c)). Moreover, fecal K8 levels around the 21st day after NEC onset had no significant difference compared with non-NEC (Ctrl1) group (68.44 ± 69.54 vs. 13.36 ± 7.96 *μ*g/ml, *p* = 0.15) (Figure 2(d)).

**Figure 3:**
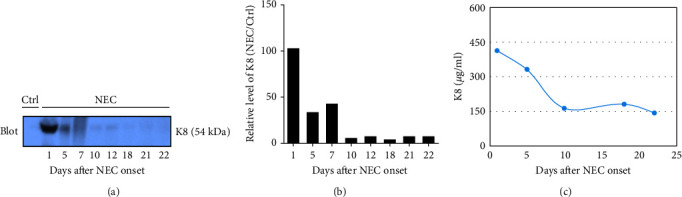
Time course of serial fecal K8 levels in an NEC neonate: (a) fecal K8 levels were detected using Western blot. Fecal K8 from a non-NEC neonate (one from the Ctrl1 group) was used as a Ctrl; (b) quantification of blots was made using ImageJ program; (c) fecal K8 levels were detected using a modified ELISA protocol. The results showed that the K8 level is highest on the first day after NEC onset. One week later, it declined to one-third of the initial level, and then persisted at a very low level from the tenth day after NEC onset. Note that fecal K8 is a potential biomarker for NEC diagnosis or monitoring the effectiveness of clinical resolution.

**Figure 4:**
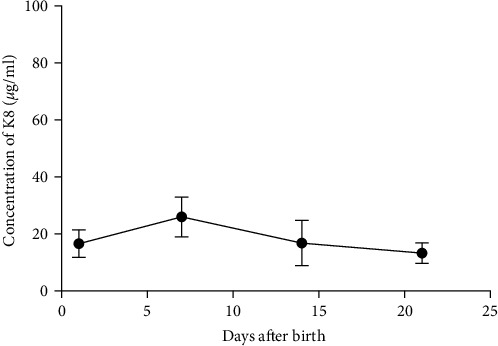
Serial fecal K8 of non-NEC neonates. To vertify the stability of K8 in non-NEC neonates after birth, we tested fecal K8 of non-NEC neonates once a week for 4 weeks. Fecal K8 levels in non-NEC preterm neonates were detected using the described modified ELISA protocol at different time points after birth (mean ± SEM) (*n* = 5). The results showed that serial fecal K8 of non-NEC neonates is stable at a low level for several weeks after birth.

A similar result was also seen in fecal calprotectin levels, which were significantly higher at the onset of NEC (*p* = 0.046) (Figure 2(e)) compared to detected levels at the time of disease resolution. However, WBC levels were not statistically different between the NEC onset group and NEC 21 days after onset group (*p* = 0.182) (Figure 2(f)). The serial change in fecal K8 levels at different days after NEC onset was detected using WB and the described modified ELISA protocol. The results of WB showed that the K8 level is highest on the first day after NEC onset. One week later, it declined to one-third of the initial level, and then persisted at a very low level from the tenth day after NEC onset (Figures 3(a) and 3(b)). Moreover, the serial fecal K8 levels detected by ELISA in the NEC group had a similar trend to the pattern that demonstrated semiquantitatively by WB (Figure 3(c)). To rule out that the trends in K8 described above are natural changes over time after birth, we tested fecal K8 of non-NEC neonates once a week for 4 weeks. The results showed that serial fecal K8 of non-NEC neonates is stable at a low level for several weeks after birth (range 3.06–39.45 *μ*g/ml) (Figure 4).

### 3.4. The Specificity of Fecal K8 Is Higher than Calprotectin for Detecting Intestinal Injury Caused by NEC

**Figure 5:**
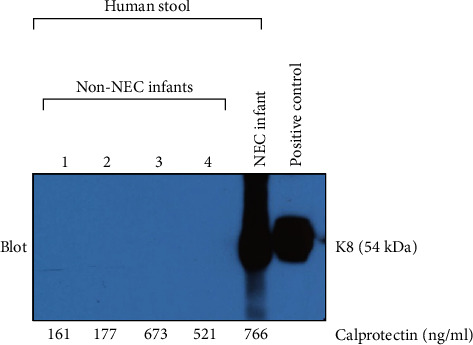
Western blot results of fecal K8 from healthy or NEC infants. All non-NEC infants with either low (161 and 177 ng/ml) or high (673 and 521 ng/ml) fecal calprotectin had a negative fecal K8 (lanes 1–4). Positive fecal K8 was identified in NEC infants (lane 5). HT29 cells are used as a positive control (lane 6). The results showed that fecal K8 has a higher specificity for NEC than calprotectin.

To compare the specificity of fecal K8 and calprotectin for detecting intestinal injury caused by NEC, we also measured calprotectin levels in the same four non-NEC infants. Two of them had a low level of fecal calprotectin (160.66 and 177.28 ng/ml), and the other two had a high level of fecal calprotectin (673.11 and 520.76 ng/ml). However, Western blot (WB) analysis showed that all non-NEC infants with either low or high fecal calprotectin had a negative fecal K8. Positive fecal K8 was identified in NEC infants. HT29 cells were used as a positive control (Figure 5).

## 4. Discussion

An accurate and specific diagnostic measure of NEC remains a significant unmet need to accurately differentiate NEC as a cause of significant GI injury and institute appropriate therapeutic measures. With continued study and further advancement in molecular diagnostic modalities, several candidate NEC biomarkers identifiable in stool, urine, and serum have been proposed with varying diagnostic accuracy [11–13]. Until now, no specific fecal biomarkers have been identified that quantitatively reflect intestinal injury and its resolution associated with NEC and further independently differentiate NEC injury from nonspecific markers of enteric inflammation caused by non-NEC etiologies. When NEC occurs, the epithelial cells of the affected small intestine (typically ileum) and colon undergo death by necrosis or apoptosis. These epithelial cells shed from the intestinal wall and are excreted with the stool. Thus, an epithelial cell (enterocyte and colonocyte)-specific molecule detectable using quantitative assays amenable to clinical utility (e.g., ELISA) may serve as a diagnostic modality amenable to clinical validation for intestinal injury associated with NEC.

K8 is an abundant cytoskeletal protein that associates noncovalently with its partners K18/K19 to form the IF cytoskeleton of intestinal epithelial cell [14]. Thus, fecal K8 is cell-type specific and only appears in clinical samples (stool) upon cellular dissolution as seen in epithelial necrosis. Moreover, K8 was found to be upregulated in the small intestine of NEC patients [15]. This change may increase the difference in fecal K8 levels between NEC and normal infants. Taken together, fecal K8 has the potential to be a marker of NEC. The current commercial ELISA kits are mainly used to detect the soluble form of K8, while in fact, 95% of K8 in the filamentous cytoskeleton is insoluble [16]. Although a combination of the strong detergent SDS with Triton X-100 can be used to solubilize some membrane proteins for subsequent detection using sandwich ELISA [17], we found that all keratins, including K8, are difficult to be solubilized. Thus, we endeavored to develop a novel modified ELISA protocol to detect the majority of fraction of insoluble fecal K8. In this study, we configured a new protocol by measuring the remaining amount of free unbound primary antibody after antigen–antibody reaction to assess the total level of both insoluble and soluble K8. The principle of this assay is similar but with distinct and important differences from a competitive ELISA since there is no real-time competition event during the process of detection for the majority of insoluble protein K8.

To ensure comparability of results and control for development or clinical exposure-dependent changes between NEC and control groups, we carefully selected non-NEC neonates with a similar GA as the NEC group. Moreover, fecal samples of the GA-matched control group were chosen on a similar postnatal day to match samples of NEC infants at the time of disease onset. Accordingly, there was no significant difference in birth weight, Apgar score, and enteral feeding between these two groups. These results suggested that the samples from these two groups were well-matched at baseline. Our results further showed that there were higher levels of K8 at the onset of NEC compared with non-NEC neonates, but levels in NEC-affected newborns gradually decreased and returned to near-normal levels upon disease resolution, allowing each patient sample series to serve as baseline and disease comparators. Importantly, our results also showed that there were higher levels of fecal K8 2 days before the clinical diagnosis of NEC compared with the non-NEC group. This result is especially intriguing since it suggests that indeed mucosal injury may be elevated in certain cases before overt clinical signs. If this result can be validated in a greatly expanded cohort, it is plausible that the K8 fecal test may broaden the current clinical window for disease prevention or provide an opportunity to limit NEC progression and prevent fulminant disease, signified by full thickness and irreversible injury, typically considered as Bell stage III disease. Taken together, these results suggest that fecal K8 may serve as a biomarker for early identification of intestinal injury of NEC.

Calprotectin, a calcium- and zinc-binding protein, is present in the stool with levels correlating with the fecal granulocyte counts [18]. In recent years, many scholars have observed that fecal calprotectin levels increase in infants with NEC and have, therefore, suggested that serial measurements may be useful as a noninvasive prognostic marker for the progression of the disease [19, 20]. However, calprotectin is an inflammatory marker, not specific to NEC, and has been associated with significant individual variation in different ages [21]. In addition, some other authors have found that there is no difference in fecal calprotectin levels between the NEC and control group. Moreover, in the NEC group, levels of calprotectin did not rise before clinical symptoms. Accordingly, these authors concluded that significant individual variation associated with fecal calprotectin renders it not conducive to use for early diagnosis or prediction of NEC in high-risk neonates [22]. In this study, we show that fecal calprotectin levels are significantly higher at the onset of NEC compared with their levels at 21 days after NEC onset. This result suggested that calprotectin may be used to assist in determining the severity of inflammation in an NEC patient during treatment. However, high fecal calprotectin levels were also identified in our non-NEC group (Figure 5, lanes 3 and 4). All non-NEC infants with either low or high fecal calprotectin had a negative fecal K8. These results suggest that fecal K8 is a more specific marker for intestinal injury in NEC in comparison to calprotectin.

WB is a recognized method for protein detection. The current WB results showed that the K8 level is highest on the first day after NEC onset, and then declines to one-third of its initial level 1 week later. These changes are due to the improvement of the patient's condition after treatment according to symptoms and WBC results. Moreover, the serial fecal K8 levels detected by our modified ELISA protocol in the NEC group had the same trend as the WB result. Together, these results suggest that this modified ELISA protocol is suitable for the detection of total protein K8. Levels of fecal K8 of non-NEC neonates remain stable at a low level from birth without a significant increase or decrease over time in the first several weeks of life. Taken together, our results suggest that fecal K8 is a strong candidate for clinical validation as a biomarker of NEC-associated intestinal injury.

A limitation of this study is the small sample size. Moreover, serial samples were not available for all infants in the NEC and control group at identical time points. In recognition of the dramatic changes that occur in the first several weeks of newborn life that may have affected the results, including gestational age, DOL, degree of enteral feeding, and gut colonization, the initial cohorts in this case–control study were carefully selected by multimatching parameters from a larger number of neonates to minimize the effect of potential confounding variables. Moreover, longitudinal sampling from the same patient in the case and control was utilized to account for some interpatient variability and to capture disease course associations. Another limitation is the coating antigen used in this study, which is HT29 cell lysate containing K8 but not commercially available purified K8 proteins. Since K8 is an abundant cellular protein in HT29 cells, there is no issue that occurred in the present ELISA; however, it should be cautious to use other epithelial cells for such purpose.

In conclusion, the modified ELISA protocol developed herein is suitable for the detection of total K8, including the majority of insoluble forms in stool in the clinical setting. Fecal K8 significantly increases in premature infants with NEC and may serve as a noninvasive and specific marker for intestinal injury in NEC. This biomarker test may be suitable as a stand-alone biomarker of NEC upon further clinical validation studies.

## Figures and Tables

**Table 1:** Clinical characteristics of the study neonates.

Characteristics	NEC (*n* = 5)	Ctrl1 (*n* = 5)	*P*-value	Pre-NEC (*n* = 5)	Ctrl2 (*n* = 5)	*P*-value
Gestational age (weeks), mean ± SD	29.1 ± 6.4	30.1 ± 1.5	0.76	27.2 ± 2.2	28.3 ± 2.9	0.53
Body weight (g), mean ± SD	1,395.4 ± 956.2	1,216 ± 344.6	0.70	856.0 ± 258.2	1,260.0 ± 654.0	0.23
Male sex, *n* (%)	4 (80%)	3 (60%)	1.0	1 (20%)	1 (20%)	1.0
DOL at NEC/control sampling (days), mean ± SD	22.2 ± 14.3	21 ± 2.3	0.86	17.4 ± 5.7	10.2 ± 4.6	0.06
Apgar score, mean ± SD						
Apgar 1 min	4.2 ± 2.3	3.4 ± 2.6	0.62	2.2 ± 2.2	6.8 ± 1.6	0.008
Apgar 5 min	7 ± 2	6.4 ± 2.2	0.66	4.8 ± 2.2	7.2 ± 1.9	0.15
Parenteral nutrition, *n* (%)	5 (100%)	5 (100%)	1.0	4 (80%)	3 (60%)	1.0
Duration (days), mean ± SD	43.6 ± 33.9	11.2 ± 7.6	0.1	13.5 ± 9.6	4.0 ± 1.0	0.09
Age at initiation of enteral feeds (days), mean ± SD	5.8 ± 3.9	2.4 ± 1.7	0.13	3.2 ± 2.5	1.4 ± 0.5	0.12
Enteral feeding, *n* (%)						
Breast milk	5 (100%)	5 (100%)	1.0	5 (100%)	5 (100%)	1.0
Formula	4 (80%)	2 (40%)	0.52	3 (60%)	3 (60%)	1.0
Both	4 (80%)	2 (40%)	0.52	3 (60%)	3 (60%)	1.0
Length of NICU stay (days), mean ± SD	83 ± 42.9	22.4 ± 19.5	0.03^*∗*^	129.6 ± 71.9	69.2 ± 28.8	0.17
Death, *n* (%)	1 (20%)	0 (0%)	1.0	1 (20%)	0 (0%)	1.0

^*∗*^*p* < 0.05, NEC and Ctrl1 groups are from Lucile Packard Children's Hospital Stanford; pre-NEC and Ctrl2 groups are from Washington University School of Medicine.

## Data Availability

Data will be made available on request.
